# Molecular Epidemiology and Evolution of West Nile Virus in North America

**DOI:** 10.3390/ijerph10105111

**Published:** 2013-10-16

**Authors:** Brian R. Mann, Allison R. McMullen, Daniele M. Swetnam, Alan D. T. Barrett

**Affiliations:** Department of Pathology, Sealy Center for Vaccine Development, Center for Biodefense and Emerging Diseases, Center for Tropical Diseases, Institute for Human Infections and Immunity, University of Texas Medical Branch, Galveston, TX 77555, USA; E-Mails: brmann@utmb.edu (B.R.M.); xav8@cdc.gov (A.R.M.); dmswetna@utmb.edu (D.M.S.)

**Keywords:** West Nile virus, molecular epidemiology, evolution, phylogenetics

## Abstract

West Nile virus (WNV) was introduced to New York in 1999 and rapidly spread throughout North America and into parts of Central and South America. Displacement of the original New York (NY99) genotype by the North America/West Nile 2002 (NA/WN02) genotype occurred in 2002 with subsequent identification of a novel genotype in 2003 in isolates collected from the southwestern Unites States region (SW/WN03 genotype). Both genotypes co-circulate to date. Subsequent WNV surveillance studies have confirmed additional genotypes in the United States that have become extinct due to lack of a selective advantage or stochastic effect; however, the dynamic emergence, displacement, and extinction of multiple WNV genotypes in the US from 1999–2012 indicates the continued evolution of WNV in North America.

## 1. Introduction

West Nile virus (WNV; *Flaviviridae*: *Flavivirus*) is an “Old World” virus which re-emerged in the mid-1990s to international concern as a major public health threat. The virus was originally isolated in 1937 from a febrile woman in the West Nile district of Uganda during routine surveillance for yellow fever virus (YFV) [1]. However, infection was not linked to overt clinical disease until the 1950s when epidemics in Israel and Egypt stimulated detailed studies on the virus and serological distribution of WNV-specific antibodies in northern and Sub-Saharan Africa [2,3,4]. Sporadic outbreaks of febrile disease occurred throughout the 1950s–1980s in Africa, Australia and the Middle East with larger outbreaks in Israel (1951–1952, 1957, and 1962), France (1962–1965) and South Africa (1974 and 1983–1984) which were self-limited with few confirmed cases of neuroinvasive or other clinical disease, including some cases of hepatitis in South Africa [5,6]. Incidence of severe WNV-related disease in both humans and equines gained momentum in the mid-1990s with epidemics in northern Africa, the Middle East, and central Europe: Algeria (1994 and 1997), Morocco (1996), Romania (1996), Tunisia (1997), Russia (1999), Israel (1998–2000) and France (2000) [5,6]. In particular, the 1996 Romanian epidemic marked the first human epidemic associated with significant incidence of encephalitic disease.

Emergence of WNV in the Western hemisphere in 1999 resulted in its rapid expansion in naive enzootic transmission cycles throughout North America and into Central America with occasional evidence of viral isolates, RNA, and/or seroconversions in some areas of South America. On-going surveillance cohorts in the United States (US) have provided significant insight into the natural transmission dynamics, ecology, and evolution of WNV over the past decade. Cumulative efforts since 1999 estimate over 3 million human WNV infections in the US with >780,000 WN fever cases, 16,000 neurologic disease (WNND) cases, and over 1,500 associated fatalities [7]. Similar to the US, recent WNV epidemics in Europe have undergone a paradigm shift with unprecedented levels of mortality and human neurologic disease during outbreaks in Hungary (2003–2005), Greece (2010–2013), Russia (2007 and 2011–2013), Austria (2008–2009), Romania (2010), and Italy (2008–2009 and 2011–2012) [8]. Possible explanations for this shift in global WNV disease are multi-factorial and are dependent on both host and environmental stimuli (discussed in other articles within this special issue); this review will focus on the emergence, displacement, and extinction of several US genotypes to discuss potential genetic dynamics driving the evolution of WNV in North America.

## 2. Global WNV Phylogenetics: Derivation of a Lineage

**Figure 1:**
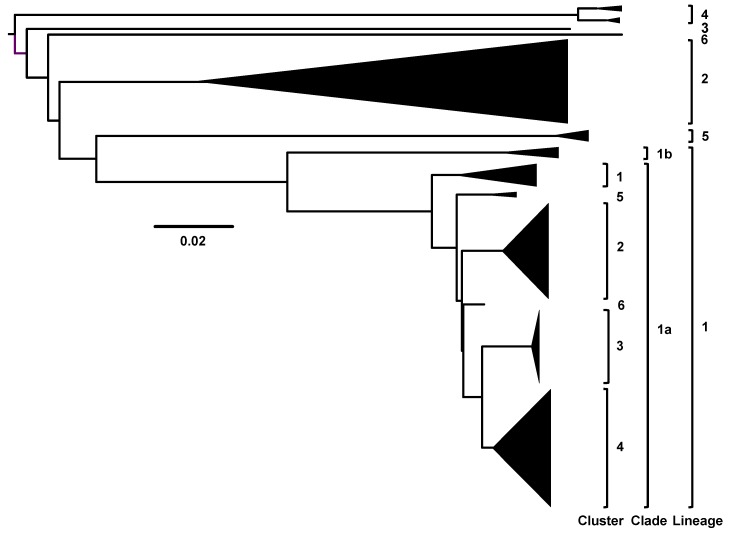
Neighbor-joining phylogenetic tree, using condensed, simplified branches that depicts the Lineages 1–6 of West Nile virus. Indicated phylogenetic groupings are defined based upon the genetic distance (*i.e*., % nucleotide divergence) for isolates which cluster within Lineages 1–6 (≥20%), Clades 1a and 1b (12.7%–20.8%) of Lineage 1, and Clusters 1–6 (≤5.4%) of Lineage 1a. Lineages 1 and 2 are the most geographically dispersed and include both endemic and epidemic strains associated with outbreaks of neurologic disease in humans, horses and birds in the Americas, Europe, Africa and the Middle East. Strains circulating in the Americas belong to Cluster 4 of Lineage 1a, as defined by May *et al*. (2011) [10].

West Nile virus has been designated into at least five distinct Lineages (1–5) based on in-depth phylogenetic analyses of published sequences in six of the seven continents (except Antarctica) collected from 1937 to the present (Figure 1; Koutango virus is indicated as a possible Lineage 6) [9,10]. Historically, Lineage 2 has circulated in sub-Saharan Africa and Madagascar in local endemic transmission cycles with limited evidence of epidemic transmission and was associated with less severe or non-neuroinvasive clinical disease in humans. However, recent epidemics in Greece, Italy, Romania, and South Africa have provided evidence of severe human neurologic disease associated with Lineage 2 WNV isolates. These epidemic isolates cluster with the non-pathogenic ancestral Lineage 2 strains [11,12,13,14,15,16,17,18]. Lineage 3 consists of a single 1997 isolate collected from a WNV-positive mosquito pool in Austria, designated Rabensburg virus (RabV), which shares 75%–77% nucleotide and 89%–90% amino acid identity with published Lineage 1 and Lineage 2 isolates [10,12,19,20]; Lineage 4 consists of several Russian isolates identified in circulation since 1988 [21]; Lineage 5 is composed of 13 Indian isolates identified from 1955–1982 that demonstrate 74%–78% nucleotide divergence from Lineages 1–4 [10,22]. Despite global epidemic circulation of Lineages 1 and 2, the epidemiological significance of Lineages 3–5 remains unclear.

Lineage 1 constitutes the largest WNV lineage with world-wide distribution of isolates further classified into two distinct clades: 1a and 1b [9,10]. Clade 1b contains Kunjin virus (KUNV) isolates in circulation in Australasia [10]. Clade 1a contains most of the Lineage 1 isolates with further subdivision into six discrete clusters based on conserved genotypic signatures [10,23,24]. Cluster 1 consists of isolates from Northern Africa (1951–1976), Israel (1953), India (1968) and Portugal (1971). Cluster 2 contains more recent isolates from Romania (1996), Morocco (1996 and 2003), Kenya (1998), Italy (1998 and 2008–2011), Russia (1999–2000), France (2000), Portugal (2004) and Spain (2007), which have been further sub-divided into the Mediterranean and Eastern European subtypes, as defined by the presence of a conserved NS1-A70S substitution in all Mediterranean subtype isolates (Table 1) [10,25,26,27,28,29]. Cluster 3 includes 1995–2005 isolates from the Astrakhan region of Russia that encode two substitutions: NS1-S99P and NS2A-A224T, which are unique to this clade. Cluster 5 is composed 1965–1979 Central African isolates, and cluster 6 contains three isolates from Nigeria (1965), the Central African Republic (1967), and Senegal (1979) [10,30]. Cluster 4 includes isolates from Tunisia (1997), Israel (1998 and 2000), Hungary (2003), and the Americas (1999–present) with conserved expression of both the E-T126I and NS4A-V85A substitutions. Furthermore, divergent evolution of a proline (residue 249) in the NS3 protein (linked with virulence in birds) is lineage-specific: Lineage 2 (H), Lineage 3 (N), Lineage 4 (T), and Lineages 1a (T/H/A), 1b (A), and 1c (T) (Table 1) [10,31].

**Table 1:** Global evolution of WNV Lineage 1a: conserved amino acid substitutions ^1^.

Lineage/Clade	Cluster	Genotype	E			NS1				NS2A			NS3			4A		4B	NS5		
			126	159	291		70	99	206		103	224		175	249		85	11	274	314	898
			T	I	K		A	S	L		A	A		I	P		V	S	S	K	T
**1a**	**1**	-	·	·	·		·	·	·		·	·		·	T		·	N	·	·	·
	**2**	Eastern European	·	M	·		S	·	F		V	·		·	·		·	·	·	·	I
		Mediterranean	·	M	·		S	·	·		·	·		·	T		·	·	·	·	·
	**3**	-	·	·	·		·	P	·		·	T		·	·		T	·	·	·	·
	**4**	-	I	V	·		*	·	·		V	·		·	·		A	·	·	·	·
		NY99	I	V	·		*	·	·		V	·		·	·		A	·	·	·	*
		SE Coastal Texas	I	V	·		*	·	·		V	·		·	·		A	·	·	·	·
		NA/WN02	I	A	R		*	·	·		V	·		·	·		A	·	·	*	·
		SW/WN03	I	A	R		*	·	·		V	·		·	·		T	·	·	R	·
		MW/WN06	I	A	R		*	·	·		V	·		·	·		A/I	·	·	*	·
	**5**	-	·	·	·		·	·	·		·	·		V	·		·	·	T	·	·
	**6**	-	·	·	·		·	·	·		·	·		·	·		·	·	·	·	·
**1b**	**-**	-	·	·	·		·	·	·		·	·		·	A		·	·	·	·	·
**2**	**-**	-	·	·	*		·	A	·		·	·		·	H		·	N	·	·	·

^1^ E, envelope; NS, nonstructural; 4A, NS4A; 4B, NS4B; Summary of amino acid changes in the WNV genome (shaded text) relative to the consensus sequence which define specific clusters or genotypes in Lineage 1, Clades a and b, or Lineage 2; Dots indicate no difference from consensus (top); Asterisks (*) indicate the presence of the indicated amino acid change in some but not all isolates.

## 3. Introduction of WNV into North America: NY99 Genotype

Initial isolation of WNV in the Western hemisphere followed a self-limited outbreak of 62 human encephalitis cases in the New York metropolitan area concurrent with 25 equine cases (9 fatal) and extensive mortality in local bird populations [32,33]. In 2000, detection of WNV-positive dead birds (in particular *Corvus* spp.) and *Culex* spp. mosquito pool isolates in New York (NY), New Jersey (NJ), Connecticut (CT), and Maryland (MD) confirmed the expansion of WNV throughout the northeastern US [33,34,35]. From 2000–2006, WNV spread down the eastern coast and across the continental US with subsequent detection in all 48 contiguous US states by 2004. National and international surveillance campaigns have also confirmed WNV circulation in regions of Canada, the Caribbean, Central America and South America [34,36,37,38,39].

**Figure 2:**
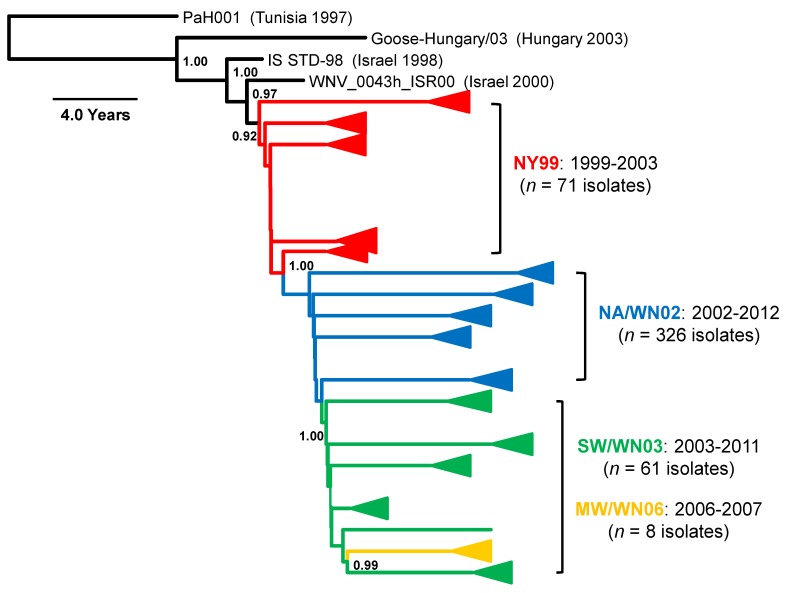
Bayesian coalescent phylogenetic tree depicting the simplified distribution of the North American WNV genotypes within Lineage 1a Cluster 4. Posterior probabilities (≥0.92) support the emergence of at least four unique WNV genotypes and/or clusters in North America from ancestral North African strains: NY99 (red), NA/WN02 (blue), SW/WN03 (green), and MW/WN06 (gold). Both the number and temporal range are indicated for the isolates which cluster within each genotype or cluster. Scale bar, divergence time in years.

The homogeneous virus population that emerged from the original expansion of WNV in the eastern US during the initial epidemic was termed the “NY99 genotype” based on the original isolation in the New York metropolitan area in 1999 (Figure 2). Complete genomic sequencing of the prototype NY99 genotype isolate (NY99-flamingo382-99 (also NY99), AF196835) cultured from a dead Chilean flamingo (*Phoenicopterus chilensis*) in the Bronx zoo in parallel with envelope (E) gene amplicons from *Culex* spp. mosquito pools and two fatal human cases confirmed the circulation of WNV in local enzootic transmission cycles [32]. Paired E-glycoprotein antigenic mapping and phylogenetic analyses of this prototype isolate revealed 99.8% sequence identity with a Lineage 1a Cluster 4 1998 Israeli domestic goose isolate (IS-98 STD, AF481864). Limited sequence similarity (≤96.9%) relative to other published epidemic Lineage 1a Cluster 2 (European) and Lineage 1b (Kunjin) strains further supported the initial introduction of WNV into the US from the Middle East or surrounding region [10,32]. However, there is no conclusive evidence of how WNV was introduced into the US or the exact location from which the virus originated. In a recent 2011 paper, May *et al*. [10] proposed the indirect role of the 1998 Israeli isolate in the initial North American outbreak; inclusion of additional European and sub-Saharan African isolates supported the independent initiation of both the Israeli and New York outbreaks upon introduction of a common progenitor strain from an unknown location in northern Africa.

In response to the initial 1999 New York epidemic, multiple WNV surveillance cohorts have monitored its spread across the US. Initial studies focused on the molecular evolution of the pre-membrane/membrane (prM/M) and E protein genes. In particular, surveillance cohorts in CT and NY from 1999–2001 confirmed the *in situ* evolution of the homogenous NY99 genotype in the northeastern US with limited genetic change (<0.18% nucleotide divergence) encoding synonymous mutations that were not fixed within the population [33,34,35,40,41]. Rapid adaptation of WNV to local, naïve *Culex* spp. mosquito and wild bird populations fueled expansion of the original NY99 genotype from the initial 1999 epicenter via migration routes across the continental US, north into Canada, and south into Central and South America [34,36,37,38,39].

## 4. Southeastern Coastal Texas Genotype

Upon introduction of WNV into Texas in 2002, a divergent population of seven uniform isolates from the coastal region of southeast Texas (termed the Southeastern Coastal Texas genotype) (Figure 2) was identified [42]. Follow-up studies of this unique clade confirmed five unique amino acid substitutions across the encoded polyprotein sequence (E-T76A, NS1-E94G, NS2A-V138I, NS4B-V173I and NS5-T526I) supporting its designation as a novel WNV genotype (as exemplified by the Kuritz isolate (also known as TVP8533; AY289214)) [43,44]. However, since 2002, there have been no isolates identified belonging to this genotype suggesting its extinction.

## 5. North America/WN 2002 (NA/WN02) Genotype

Despite limited initial public health impact, clinical incidence of WNV infection peaked between 2002–2003 with a combined total of 5,812 WNND cases and 548 fatalities [45]. Phylogenetic analyses of respective 2001–2004 North American isolates confirmed the displacement of the original NY99 genotype in 2002 with a heterogeneous pool of isolates, termed the North American (NA/WN02) genotype (Figure 2), characterized by 13 conserved nucleotide changes and an encoded substitution, V159A, in the E protein (Table 1) [41,46,47]. NA/WN02 genotype isolates exhibited an average 0.24% nucleotide and 0.09% amino acid divergence from NY99 and up to 0.58% nucleotide divergence with other NA/WN02 isolates [47]. The novel E-V159A substitution has been linked to a reduced extrinsic incubation period in *Culex* spp. mosquitoes [46,48]. In effect, extinction of the original NY99 genotype is attributed to the more efficient dissemination of NA/WN02 isolates in the mosquito vector based on still unclear species- and population-specific dynamics [48,49]. In addition, annual reintroduction and local over-wintering of virus populations have been proposed as additional potential drivers of WNV evolution and diversification on both a local and national scale [50,51]. Overall, due to evidence of transient local genotypes, such as the Southeast Coastal Texas genotype (which lacked the E-V159A substitution), fixation of the NA/WN02 genotype in North America supports the selective advantage of the diverse, dynamic virus populations within this dominant genotype [42,43,44].

## 6. Southwest/WN 2003 (SW/WN03) Genotype

**Figure 3:**
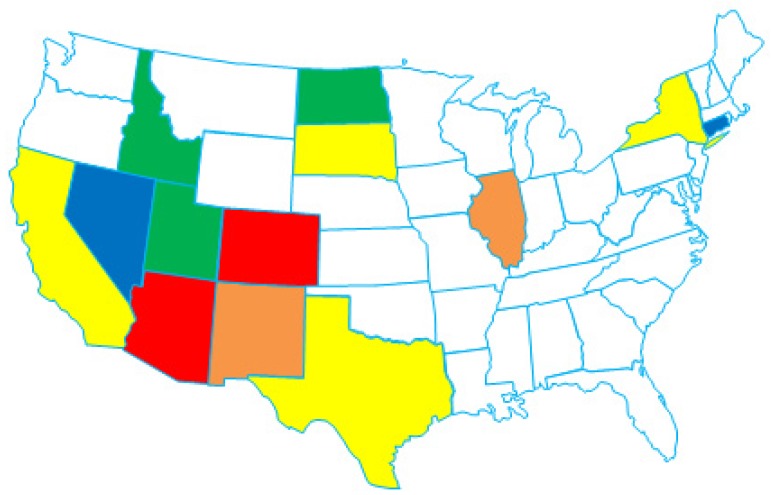
Map of the United States showing states with the SW/WN03 genotype. Colors refer to year that the SW/WN03 genotype was first identified from isolates collected in a particular state. Red = 2003, orange = 2004, yellow = 2005, green = 2006, and blue = 2008.

Following emergence of the NA/WN02 genotype, progressive declines in clinical WNV disease from 2004–2011 have been correlated with regional homeostatic WNV populations and a decreasing national growth rate consistent with now endemic WNV circulation in the US [41,45,52,53]. Despite these trends, continued WNV surveillance efforts in the southwestern US identified a pool of 2003–2009 isolates with novel phylogenetic relationships relative to the dominant NA/WN02 genotype. Confirmation of 13 unique nucleotide changes defined the emergence of the additional southwestern US (SW/WN03) genotype in 2003 with positive selection for both the NS4A-A85T and NS5-K314R substitutions in multiple isolates (Figure 2) [54]. Both the NA/WN02 and SW/WN03 genotypes co-circulate in the US to date; however, the majority of the distribution of the SW/WN03 genotype remains restricted to the southwestern US with occasional isolates found in other states (Figure 3).

## 7. Midwest/WN 2006 (MW/WN06 Cluster)

Ongoing surveillance efforts with more recent 2006–2011 isolates have also identified a novel cluster of WNV isolates derived from human blood donors and birds from Idaho and North Dakota collected during 2006–2007 (termed the MW/WN06 cluster, Figure 2) within the SW/WN03 genotype [55]. In addition, 27 of the 29 human isolates in these phylogenetic groups encode the characteristic SW/WN03 genotype NS4A-A85T substitution with 50% of these isolates also encoding the NS5-K314R substitution [54,55]. Interestingly, despite fixation of E-V159A and selection of NS4A-A85T in North American WNV populations, both positions exhibit genotypic variation and independent evolution on a global scale. In particular, although all immediate ancestors of Lineage 1a Cluster 4 demonstrate a valine at position 159 in the envelope (E) protein, all other WNV isolates express either a methionine or consensus isoleucine (Table 1). NS4A-85T is also fixed in all published Lineage 1a Cluster 3 isolates [10].

## 8. 2012 United States Epidemic

Endemic enzootic circulation of WNV in the US since 2006 has coincided with a dramatic decline in the confirmed incidence of clinical disease with a total of <1,100 reported WNND cases between 2008–2011 [45]. By comparison, WNV transmission in the recent 2012 US epidemic demonstrated a significant divergence from the national *status quo*. Overall, in 2012, the US witnessed 5,674 confirmed WNV disease cases with 2,873 reports of WNND and 286 fatalities for a national incidence of 0.92 per 100,000 population, comparable to peak 2003 statistics [45,56,57]. National attention focused on the Dallas/Fort Worth, Texas metropolitan area, which represented >29% of the US public health burden and >50% of all reported neurologic cases [45,56]. In-depth phylogenetic analyses identified the co-circulation of three independent genetic groups in resident birds and *Culex* spp. mosquito pools collected from the greater Houston and Dallas/Fort Worth, Texas regions, which had 0.41–0.72% nucleotide divergence from NY99 [58,59]. Despite isolation of these isolates in the southwestern region of the US, all 2012 isolates clustered within the NA/WN02 genotype with published 2006–2009 NY and CT isolates. None of these isolates expressed the NS4A-A85T and NS5-K314R substitutions of the SW/WN03 genotype; however, there was evidence for positive selection of both NS2A-I52T (in all sampled isolates) and NS5-314 substitutions in independent follow-up studies [58,59]. These surveillance studies highlighted the continued evolution of WNV in the US; significantly, no correlation was identified between regional differences in WNV genetics and the *in situ* rise in encephalitic disease.

## 9. Mexico, Central America, and South America

Despite current endemic circulation of WNV in the US, incidence of clinical encephalitic disease in Mexico is restricted to 8 confirmed cases between 2003–2009 in the northern Mexican States of Chihuahua, Sonora, and Nuevo Leon [36,60,61]. National screening campaigns of local equine populations from 2002–2007 identified up to 62.5% seroprevalence of WNV-specific antibodies limited to the northern and southeastern Mexican States with little clinical disease [62,63,64,65]. In effect, current evidence supports the introduction of WNV into Mexico from the US in two (or more) independent events: (1) bird migration from the southeastern US into the Yucatan Peninsula prior to 2003 and (2), several independent reintroduction events on the US-Mexican border since 2003 [36].

Isolation of the prototype TM171-03 (AY660002) raven isolate from southeastern Tabasco State in 2003 supports the initial introduction of WNV into the Yucatan Peninsula from migratory bird populations on the Pan-American Atlantic flyway [36]. TM171-03 clusters within the now extinct NY99 genotype with 0.42% nucleotide divergence from the prototype NY99 isolate; furthermore, absence of the E-V159A substitution supports immigration of the TM171-03 isolate or a progenitor strain prior to emergence of the NA/WN02 US genotype in 2002 [66,67]. Isolation of 15 additional WNV isolates between 2004–2009 was restricted to the northern States of Baja California Norte, Chihuahua, Nuevo Leon, Sonora, and Tamaulipas [60,68,69]. In-depth phylogenetic comparisons of these isolates identified closer relationships with NY99 (0.22–0.54% divergence) *versus* TM171-03 (0.40–0.76%); restricted clustering within the NA/WN02 and SW/WN03 US genotypes gives further support for the independent introduction of these isolates across the US-Mexican border since 2003 [54,68,69]. Recent evidence for the dynamic WNV transmission across the US-Mexican border provides additional selective pressure for WNV evolution in the southwestern US [69].

Outside of Mexico, serological evidence of WNV exists within several countries in the Caribbean and Central America in addition to some South American regions in Argentina [38], Brazil [70], Colombia [39], and Venezuela [71]. In particular, isolation of WNV in South America remains restricted to two 2006 Argentinian cases of fatal equine encephalitis [38] and two 2008 Colombian isolates from captive American flamingoes (*Phoenicopterus ruber*) [39]. In both studies, all four isolates clustered within the NY99 genotype with increased sequence divergence to published 2003–2009 Mexican isolates. Current evidence supports the direct lineage of these isolates within the NY99 genotype; however, how these isolates immigrated into South America remains unclear [38,39]. No evidence exists for the alternative introduction of WNV into Central/South America outside the US, and no additional South American isolates have been isolated. Possible explanations for the relative absence of WNV in Mexico, Central America, and South America to date include (1) serologic cross-protection and/or competition with other endemic flaviviruses (e.g., St. Louis encephalitis, yellow fever, dengue, and mosquito-specific viruses) [72,73,74]; (2) under-reporting or clinical misdiagnosis under the dengue fever clinical umbrella; (3) a range of other potential host, environmental, and socio-economic factors.

**Figure 4:**
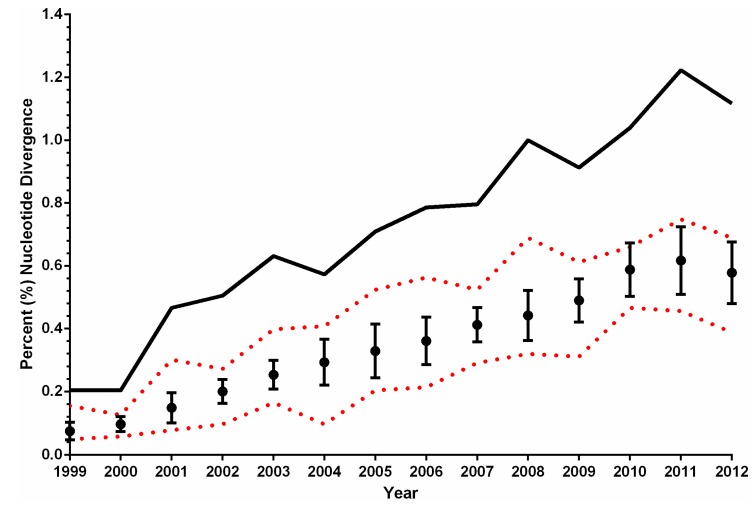
Continued evolution and genetic variation of North American WNV isolates, 1999–2012. North American WNV isolates (*n* = 454) published in GenBank between 1999–2012 were compared to the prototype NY99-flamingo382-99 isolate (NY99, AF196835) [32] to determine the average annual percent (%) nucleotide divergence (black dots). Error bars indicate standard error from mean values. Red dotted lines, respective minimum and maximum annual % nucleotide divergence from NY99. Evaluation of overall annual WNV diversity (black line) is demonstrated as the maximum annual % nucleotide divergence between all published virus sequences. Estimates for 2012 included isolates from the 2012 Texas epidemic alone [45,56].

## 10. National Outlook: Is it the End or the Beginning?

With the displacement, extinction, and co-circulation of multiple genotypes during its tenure in North America, forecasts for the evolution of WNV in the US remain conflicted. Is WNV evolution gaining momentum or coming to a halt? Initial published models predicted a mean substitution rate of 3.6 × 10^−4^ substitutions/site/year with a rapid decline in regional WNV variability following the 2002–2003 peak in human clinical incidence [52]. Studies of North American WNV isolates collected from 1999–2011 have suggested that the virus has reached genetic homeostasis in North America consistent with limited sequence variation and the lack of emergent genotypes since 2006 [41,53,55]. However, much of this evidence was based on partial genome sequences (prM/E) and only included isolates from 1999–2005. In contrast, established WNV surveillance cohorts in both Chicago, Illinois [50,75,76] and Houston, Texas [44,47,54,59] highlight the continued evolution and diversification of WNV on a fine-geographic scale on par with both national and global trends [10]. Consider Figure 4 which depicts the mean annual % nucleotide divergence of all published North American WNV isolates (*n =* 454 sequences) collected to date from the prototype NY99-flamingo382-99 (NY99) strain. From a national perspective, WNV isolates appear more diverse from original NY99 populations as time progresses. However, does this trend model evolution of a uniform national virus population or the continued microevolution of distinct WNV sub-populations on a local and/or regional scale? Calculation of the annual maximum percentage (%) nucleotide divergence between all published WNV sequences provides a more accurate approximation of WNV variability over time; as seen in Figure 4, the genetic variation of North American WNV isolates continues to increase consistent with the proposed expansion of local or regional WNV populations [50,75,76]. Overall, despite endemic circulation over the past decade, WNV continues to evolve in the United States through still unknown host and/or ecological selective pressures.

## 11. Conclusions: The Future; Interpreting Genetic Data

**Figure 5:**
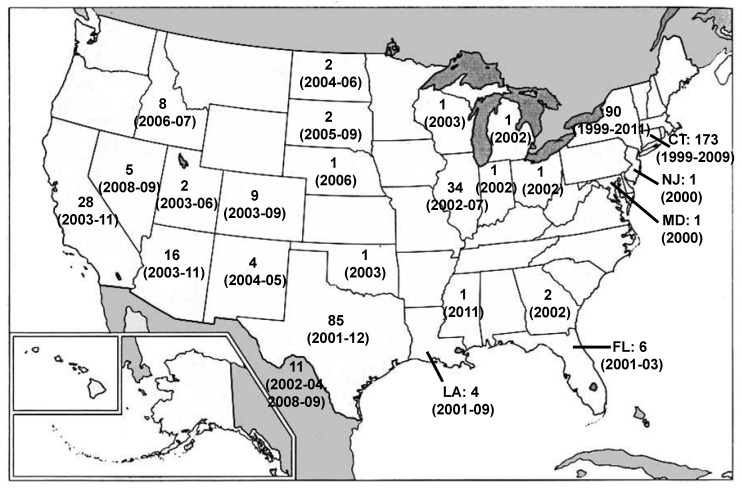
Map of the United States showing genomic sequences available by state and year.

*In silico* phylogenetic models provide an effective approach to reconstruct the ancestral lineages or “relatedness” between target virus populations; however, such approaches are dependent on the applied dataset with critical biases from non-uniform host and/or regional surveillance (termed sampling bias). Such caveats include the over-representation of WNV isolates from a few select US or Mexican States in all current North American surveillance and phylogenetic studies. Multiple cohorts have described the evolution of WNV within localized geographic regions (city and/or state) across single or multiple years including California (CA) (complete genomes: 2003–2005) [77], CT (prM/E and complete genomes: 1999–2008) [35,51], Florida (FL) (prM/E: 2003–2005) [78], Illinois (IL) (complete genomes: 2002–2007) [50,75,76], Mexico (complete genomes: 2003–2010) [36,66,67,68,69], NY (E, NS5, and 3′-UTR: 2000–2003) [33,46], Puerto Rico (prM/E: 2007) [79], and TX (prM/E and complete genomes: 2002–2012) [41,42,44,47,53,54,59,69] (see Figure 5 for available genomic sequences by state). Overall, the above studies represent >71% of all published WNV isolates (Figure 5); furthermore, the conclusions from these analyses have demonstrated the co-circulation of localized clades introduced from other North American regions on a fine-geographic scale. Ongoing surveillance cohorts in these regions continue to exhibit neutral selection of circulating WNV clades with no additional evidence of fixed genotypic mutations. However, subsequent studies of the 2012 epidemic clearly show that the situation is more complex with 2012 TX isolates clustering within the NA/WN02 genotype with published 2006–2009 NY and CT isolates [58,59].

Comprehensive surveillance and phylogenetic analysis of isolates from all US States would limit the current sampling bias attributed to current datasets (Figure 5). In addition, one central complication in the field is the correlation between genotypic variation and epidemic clinical disease. Due to the multi-factorial nature of WNV infection and disease progression, a single genetic change can alter WNV host fitness or virulence. Despite known limitations, phylogenetic and genotypic analyses have suggested that WNV virulence is multigenic with identification of several genetic determinants implicated in continued WNV evolution, which also correlate with differences in WNV phenotype in the mosquito vector, avian reservoir, or human/equine dead-end hosts [48,77,80,81,82,83,84,85].

It is clear that these factors are multi-factorial; however, elucidating the underlying factors responsible for and/or affecting this evolution has been difficult. Despite these uncertainties, evidence for evolution of this virus since its introduction is well-defined with documented multigenic viral changes in response to numerous external contributors such as the variety and distribution of virus-infected hosts, climate, and additional ecological aspects. Continued investigation of genetic changes over time is critical to understanding the spread of the virus, viral and host factors contributing to virulence and mosquito competence, and the possible future of vaccines or drug candidates. In the aftermath of the significant 2012 US epidemic, which few anticipated, continued preparation for future epidemics is critical, for which (at present) we are unable to predict.
